# Dynamically reconfigurable nanoscale modulators utilizing coupled hybrid plasmonics

**DOI:** 10.1038/srep12313

**Published:** 2015-07-20

**Authors:** Charles Lin, Amr S. Helmy

**Affiliations:** 1 The Edward S. Rogers Sr. Department of Electrical and Computer Engineering, University of Toronto, 10 King’s College Road, Toronto, Ontario M5S 3G4, Canada

## Abstract

The balance between extinction ratio (*ER*) and insertion loss (*IL*) dictates strict trade-off when designing travelling-wave electro-optic modulators. This in turn entails significant compromise in device footprint (*L*_3*dB*_) or energy consumption (*E*). In this work, we report a nanoscale modulator architecture that alleviates this trade-off while providing dynamic reconfigurability that was previously unattainable. This is achieved with the aide of three mechanisms: (1) Utilization of epsilon-near-zero (ENZ) effect, which maximizes the attainable attenuation that an ultra-thin active material can inflict on an optical mode. (2) Non-resonant coupled-plasmonic structure which supports modes with athermal long-range propagation. (3) Triode-like biasing scheme for flexible manipulation of field symmetry and subsequently waveguide attributes. By electrically inducing indium tin oxide (ITO) to be in a local ENZ state, we show that a Si/ITO/HfO_2_/Al/HfO_2_/ITO/Si coupled-plasmonic waveguide can provide amplitude modulation with *ER* = 4.83 dB/*μ*m, *IL* = 0.03 dB/*μ*m, *L*_3*dB*_ = 622 nm, and *E* = 14.8 fJ, showing at least an order of magnitude improvement in modulator figure-of-merit and power efficiency compared to other waveguide platforms. Employing different biasing permutations, the same waveguide can then be reconfigured for phase and 4-quadrature-amplitude modulation, with actively device length of only 5.53 *μ*m and 17.78  *μ*m respectively.

Efficient optical modulators constitute one of the key components in high-performance optical and data communications, signal processing, and microwave photonics^1^,^2^. Maximizing the figure-of-merit associated with modulators require the largest extinction ratio (ER) and bandwidth attainable with minimal insertion loss (IL), footprint, switching time, and power consumption. Past modulator architectures such as Si-based interferometric platforms^3^, high-Q resonant structures^4^, electro-absorption approach using compound semiconductors^5^, and plasmonic-based designs^6^ often address a subset of these modulator attributes. They are seldom able to optimize all of the relevant parameters simultaneously, dictating trade-offs and thus suboptimal performance. On the other hand, emerging data communication applications involve dense integration of electronic circuits with optoelectronic devices in two and three dimensions^7^,^8^. For these upcoming applications, true athermal operation, further device miniaturization, and improved energy efficiency of existing optoelectronic transceivers are mandatory for meeting the required specifications. New optical materials and architectures that can deliver the necessary leap in performance metrics are still lacking.

Recently, transparent conducting oxides (TCOs) such as indium tin oxide (ITO) have shown significant promise as emerging active materials for next-generation optical modulators^9^,^10^,^11^,^12^,^13^,^14^,^15^,^16^,^17^,^18^. ITO is attractive for high-speed electro-optic modulation as it exhibits high electrical conductivity^19^ and has gate-variable plasma frequency, which allows for epsilon-near-zero (ENZ) effect as well as dielectric-metal transition in the near-infrared regime^20^,^21^. Specifically, the formation of accumulation layer can occur through external gate bias. Such layer can exhibit plasma frequency and permittivity that are tunable and vary significantly from that of bulk ITO. However, a considerable bottleneck that hinders the performance of ITO-assisted optical modulation is the limited light-matter-interaction (LMI) achievable within the device footprint. This is because electrically-induced refractive index change only manifests within the locally-induced carrier accumulation layer, which is few nanometers in thickness and thus have negligible overlap with typical optical modal cross-section. For planar waveguide configuration, where the light wave interacts with ITO overlaid on top of the waveguide, device length of 10 s of *μ*m is necessary^9^. This extended device footprint in turn poses limit on potential improvement in energy efficiency. On the other hand, such footprint constraint is mitigated by utilizing ITO as a dielectric layer within plasmonic waveguides^10^,^11^,^12^,^13^,^14^,^15^,^16^,^17^,^18^. Nonetheless, while a plasmonic-based approach further enhances the mode overlap and the electromagnetic field strength across the electrically modulated ITO, the improved ER of few dB/*μ*m is accompanied by significant increase in the IL. Moreover, the aforementioned investigations have only focused on amplitude modulation and the potential for ITO-assisted phase modulation or coherent modulation have not been explored. As such, there is still a need for a compact, low-power modulator design that can better exploit the tunable properties of ITO, while averting the strict ER-IL trade-off and supporting different modulation formats.

In this work, we report a coupled-hybrid plasmonic waveguide architecture that is comprised of Si/ITO/HfO_2_/Al/HfO_2_/ITO/Si stack. The design is capable of alleviating the ER-IL trade-off and at the same time offering fJ-level power consumption as well as reconfigurable modulation modalities. In our device, the ENZ effect in electrostatically-gated ITO is utilized to manipulate the field distribution within the coupled-waveguide system. Harnessing the LMI enhancement induced by the ENZ effect, the change in permittivity within a 1 nm accumulation layer can induce strong carrier absorption as well as adversely disturb the field symmetry responsible for long-range mode propagation, rendering the otherwise low-loss waveguide highly absorptive. Utilizing different biasing configurations of the two embedded ITO layers, waveguide dispersion characteristics can then be further manipulated to enable amplitude, phase, as well as quadrature-amplitude-modulation (QAM).

## Results

### Modulator Configuration

**Figure 1:**
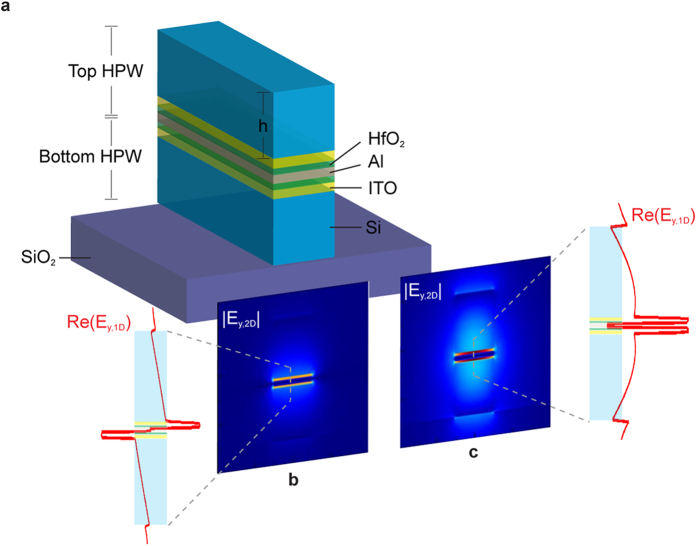
Legend: ITO-assisted coupled-HPW modulator and supported optical modes. **(a)** Schematic of the waveguide. **(b)** Simulated field distribution of the antisymmetric and **(c)** symmetric supermode supported by the waveguide at *λ* = 1550 nm (the *E*_*y*_ field component is plotted in both 1D and 2D). The mode intensity is uniformly distributed across both HfO_2_ and ITO layers. The waveguide width is 200 nm with layers of the following thicknesses: *t*_*Si*,*bottom*_ = 220 nm, *t*_*Si*,*top*_ = 240 nm, 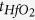 = 5 nm, *t*_*ITO*_ = 10 nm, *t*_*Al*_ = 10 nm, and 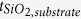 = 2 *μ*m. The carrier density of ITO is set to 1 × 10^19^ *cm*^−3^.

The proposed modulator architecture is shown in Fig. 1a. It consists of top and bottom hybrid plasmonic waveguides (HPWs) that are coupled through a common Al layer. Each HPW stack forms a metal-oxide-semiconductor (MOS) capacitor, where Al and ITO layers can be contacted. The application of gate voltage can capacitively induce an electron accumulation layer at the ITO-HfO_2_ interface that is ~1 nm in thickness, which was calculated based on Thomas-Fermi screening theory^13^. By tuning the carrier concentration (*n*_*acc*_) and thus the relative permittivity (*ε*_*r*_) of the accumulation layers in the two ITO films, the fundamental ER-IL trade-off can be addressed using the following two-step design strategy:

The first step aims to maximize the modulator ER via ENZ effect. In a HPW, the optical mode is primarily confined within the nanoscale spacer region^22^,^23^. The mode is predominantly of a plasmonic nature with only a small fraction guided through total-internal-reflection within the high-index dielectric core. Thus, placing the actively-gated ITO within the spacer layer allows for stronger optical mode overlap and light-matter interaction, leading to compact form-factor and enhanced performance^10^,^11^,^12^. More importantly, in a HPW, the field intensity and power absorbed per-unit-area is proportional to Im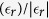 of the spacer^10^. Thus, as the local 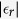 approaches zero, it is possible to draw electromagnetic energy away from the high-index Si core and the HfO_2_ spacer layer, thereby concentrating the optical field across the ITO accumulation region. Due to the strong free carrier absorption and the Ohmic loss originating from the adjacent metal layer, the mode propagation length will become greatly reduced. As a result, ENZ effect can empower refractive index change within a 1 nm accumulation layer to significantly disturb a well-guided hybrid plasmonic mode^10^,^11^,^12^.

The 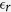 of ITO as a function of *n*_*acc*_ can be described by the Drude model (See Method)^9^. To ensure that ITO can exhibit free electron behavior instead of behaving as a Mott insulator, we assume doping level of 1 × 10^19^*cm*^−3^ for bulk ITO film that is under zero external bias^9^. Moreover, we only allow *n*_*acc*_ of up to 1.4 × 10^21^*cm*^−3^ for the 1 nm accumulation layer to prevent electric field breakdown of the HfO_2_ layer^24^. From Supplementary Fig S1, it can be observed that 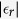 of ITO accumulation layer can vary rapidly from a maximum of 3.84 at *n*_*acc*_ = 1 × 10^19^*cm*^−3^ to a minimum of 0.57 at *n*_*acc*_ = 6.6 × 10^20^*cm*^−3^ for *λ* = 1550 nm. When biased to *n*_*acc*_ = 6.6 × 10^20^ *cm*^−3^, the enhanced LMI allows full capitalization of ITO’s absorptive nature, leading to significant OFF-state loss of 7.12 dB/*μ*m (see Supplementary Fig. S2). However, similar to other plasmonic-based, ITO-assisted modulators^11^,^12^,^13^,^14^,^17^,^18^, this is accompanied by excessive ON-state loss of 0.91 dB/*μ*m due to Ohmic dissipation. Defining the IL as the ON-state loss of the waveguide and the ER as the difference between ON-state and OFF-state losses, this modulator configuration provides IL = 0.91 dB/*μ*m and ER = 6.21 dB/*μ*m. Although 3-dB amplitude modulation only requires active waveguide length (*L*_3*dB*_) of 483 nm, the figure-of-merit (FOM), which is defined as ER/IL, for this ITO-assisted HPW is calculated to be only 6.82.

The second step aims to minimize the IL by designing athermal coupled-plasmonic structures that support long-range propagation. When the common Al layer is sufficiently thin (Fig. 1a), the mutual-perturbation of the top and bottom HPW modes will lead to the formation of transverse antisymmetric (Fig. 1b) and symmetric supermodes (Fig. 1c)^25^. The antisymmetric supermode corresponds to in-phase coupling of two HPW modes, resulting in stronger field intensity overlapping the metal and thus significant propagation loss. Conversely, the symmetric supermode is associated with destructive interference of the two HPW modes, which lowers the Ohmic loss and enables extended propagation length^26^. Therefore, utilizing the symmetric supermode as the signal carrier for modulation can significantly reduce the IL overhead. The loss characteristics of the supermodes depend sensitively on the field symmetry across the common metal layer. Specifically, IL can be minimized by manipulating the waveguide parameters such that complete field symmetry is established for the symmetric supermode when the modulator is in the ON-state. Since the mode power is distributed across the ITO and HfO_2_ layers, the 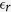 of the 1 nm ITO accumulation layer will have negligible effect on this long-range hybrid plasmonic mode in the ON-state. However, when the waveguide is biased to the OFF-state, significant optical loss will then take place due to a combination of two causes: (1) the minimization of 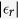 within the accumulation region leads to propagation loss enhancement similar to the case of ITO-assisted HPW. (2) the change in 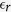 also alters the symmetry of field distribution, further increasing the mode loss. Thus, tuning the optical properties of exceedingly thin layer can render the symmetric supermode highly lossy. This approach allows us for the first time to maintain the superior ER and device footprint established with aide of ENZ effect, while minimizing the corresponding IL.

**Figure 2:**
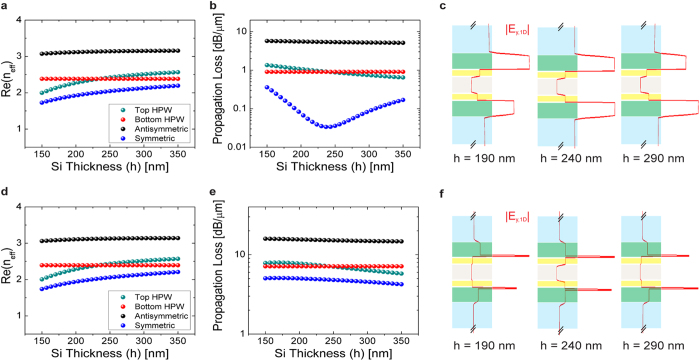
Legend: Effect of field symmetry engineering within ITO-assisted coupled-HPW structure. The mode properties of the waveguide are plotted as a function of the thickness of the top Si layer (*h*): **(a)** *n*_*eff*_ and **(b)** loss of the waveguide modes at *n*_*acc*_ = 1 × 10^19^ *cm*^−3^. Waveguide loss reaches minimum at the crossing point between the *n*_*eff*_ of the decoupled HPW modes. **(c)** 1D |*E*_*y*_| field distribution of the symmetric supermode for different Si thicknesses. The schematics are not to scale as only the field distribution near the metal is plotted. In this ON-state, optical mode is primarily confined and distributed within the low-index spacer layers (both ITO and HfO_2_). The field profile is symmetric across the metal layer at *h* = 240 nm, where waveguide loss is minimized. **(d)** *n*_*eff*_ and **(e)** loss of the waveguide modes at *n*_*acc*_ = 6.6 × 10^20^ *cm*^−3^. The loss of the symmetric supermode is no longer minimized at *h* = 240 nm. **(f)** 1D |*E*_*y*_| field distribution of the symmetric supermode for different Si thicknesses. In this OFF-state, mode power is concentrated into the ITO accumulation layers. The field profile across the metal layer becomes asymmetric at *h* = 240 nm. The results are calculated at free space *λ* = 1550 nm.

To demonstrate the effect of field symmetry engineering, the thickness of the top Si layer (*h*) is varied and the dispersion and loss properties of the supermodes supported by the example structure shown in Fig. 1a are plotted in Fig. 2. For simplicity, the thickness of other material layers are kept constant and the two ITO films are injected with the same *n*_*acc*_. The modal properties of the top and bottom HPWs are also displayed for comparison. For *n*_*acc*_ = 1 × 10^19^*cm*^−3^, *n*_*eff*_ of the top HPW increases with *h* and approaches that of the bottom HPW, eventually intersecting at *h* = 240 nm (Fig. 2a). Concurrently, the propagation loss of the symmetric supermode decreases with increasing *h*, with a minimum propagation loss of 0.03 dB/*μ*m at the crossing point (Fig. 2b). Figure 2c reveals that this crossing point corresponds to the complete symmetric field distribution that is required for a transparent ON-state. On the other hand, for *n*_*den*_ = 6.6 × 10^20^ *cm*^−3^, the loss of the symmetric supermode is no longer minimized at the crossing point (Fig. 2e) and the modal energy distribution with respect to the metal becomes slightly asymmetric (Fig. 2f). Due to the ENZ effect and the asymmetric field distribution, the waveguide is transformed into OFF-state with propagation loss of 4.86 dB/*μ*m at *h* = 240 nm (Fig. 2e). Thus, field symmetry engineering, which leads to long-range mode propagation within the waveguide, can offer ER = 4.83 dB/*μ*m, IL = 0.03 dB/*μ*m, and FOM = 161.

When compared with that of a single ITO-assisted HPW modulator, the coupled-HPW structure designed using this methodology provides a 97% reduction in the IL and a 24-fold improvement in the FOM, at the cost of only 22% reduction in the ER. This alleviates the ER-IL trade-off while maintaining a nanoscale footprint of *L*_3*dB*_ = 622 nm. Moreover, the propagation loss of the symmetric supermode at *n*_*acc*_ = 1 × 10^19^ *cm*^−3^ only increases by 0.01 dB/*μ*m when *h* deviates from the optimal value by ± 25 nm, which is ~ ± 10% of the intended dimension (Fig. 2b). This highlights how the design is non-resonant and athermal, with strong fabrication tolerance. In general, the antisymmetric supermode has *n*_*eff*_ and loss that are at least 0.8 and 7 dB/*μ*m higher than that of the symmetric supermode respectively. Thus, single-mode operation and negligible cross-coupling can be expected for this architecture. Table I provides a comparison of the performance of our design against previous reports of ITO-assisted modulators, which utilized various thicknesses for the voltage-induced accumulation layer, ranging from 1 nm to 10 nm^9^,^11^,^12^,^14^,^17^. Our device, with 1 nm accumulation layer, exhibits ER and IL comparable to plasmonic-based and dielectric-based designs respectively, thus showing significant improvement in the overall modulator FOM. Note that the FOM of our design can be further enhanced by allowing the thickness of the accumulation region to increase, reaching 641 when the entire 10 nm ITO layer undergoes change in refractive index (see Supplementary Fig. S3). It should be pointed out that the work in Ref. 18 applied a similar approach where the *n*_*acc*_ of gallium-doped zinc-oxide is tuned to perturb the symmetric supermode of a long range plasmonic structure. However, the accumulation layer thickness was taken to be 10 nm and since field symmetry effect was not exploited, their modulator IL could not be reduced to a level comparable to its dielectric counterparts.

**Figure 3:**
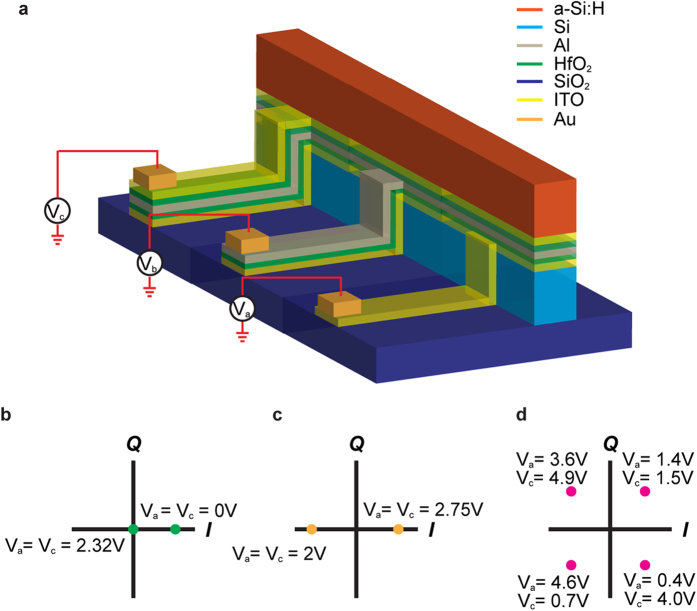
Legend: Modulator biasing configuration and associated modulation modalities. **(a)** Schematic of the biasing configuration of the ITO-assisted coupled-HPW modulator. **(b)-(d)** The biasing conditions required to achieve the constellation points for different modulation formats. Having *V*_*a*_ = *V*_*c*_ can provide either amplitude or phase modulation whereas allowing *V*_*a*_ ≠ *V*_*c*_ can support complex coherent modulation formats such as 4-QAM. The Al contact is grounded in all cases (*V*_*b*_ = 0 V).

Bias Configuration and Modes of Operation. For a single modulator to support different modulation formats, a triode-like biasing configuration that allows the ITO and Al films to be independently biased is required (Fig. 3). The proposed experimental waveguide configuration is illustrated in Fig. 3a, while the supported modulation formats and the corresponding gating conditions are shown in Fig. 3b–d. The fabrication of our multilayer structure requires electron-beam-lithography patterning in combination with lift-off process^11^,^14^. The HfO_2_ and ITO layers can deposited through RF magnetron sputtering, where the carrier concentration of as-deposited ITO can be controlled by tuning the oxygen concentration during deposition^14^. Moreover, hydrogenated amorphous polysilicon can be deposited via low temperature-PECVD and serves as the top high-index dielectric layer^27^. To minimize the disturbance to the optical mode, electrical contacts will be made through Au pads^11^,^14^ that are evaporated onto narrow strips of Al and ITO films, which have been extended several hundred nanometers away from the waveguide.

**Figure 4:**
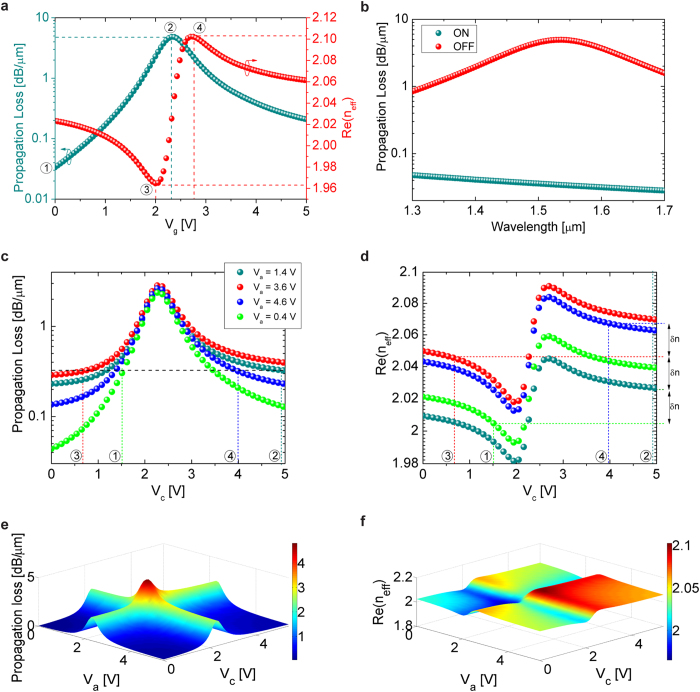
Legend: Modulation performance and capabilities of ITO-assisted coupled-HPW modulator. **(a)** Propagation loss and *n*_*eff*_ of the waveguide as a function of *V*_*g*_ (*V*_*a*_ = *V*_*c*_ = *V*_*g*_ and *V*_*b*_ = 0 V). Amplitude modulation can be achieved using biasing conditions 1 and 2 while phase modulation can be obtained using biasing conditions 3 and 4. **(b)** The propagation loss of the waveguide as a function of wavelength. The ON-state loss is controlled by the extent of field symmetry within the structure and thus exhibits broad bandwidth. The OFF-state loss is dictated by the ENZ effect and therefore exhibits narrower bandwidth. **(c)** Propagation loss and **(d)** *n*_*eff*_ of the waveguide as a function of *V*_*c*_ for different *V*_*a*_’s (*V*_*b*_ = 0). For the four labeled biasing combinations, waveguide losses are nearly identical but the corresponding *n*_*eff*_ values are separated by an uniform *δ*n of 0.0218. **(e)** Propagation loss and **(f)** *n*_*eff*_ of the waveguide as a function of both *V*_*a*_ and *V*_*c*_ (*V*_*b*_ = 0 V).

Figure 4a shows the modulator characteristics for the simplest biasing configuration where the Al contact is grounded (*V*_*b*_ = 0 V) while *V*_*a*_ = *V*_*c*_ = *V*_*g*_. The ITO films are assumed to be chemically doped to *n*_*acc*_ = 1 × 10^19^ *cm*^−3^ at *V*_*g*_ = 0 V. As expected from previous analysis, waveguide loss reaches maximum of 4.86 dB/*μ*m at *V*_*g*_ = 2.32 V (*n*_*acc*_ = 6.6 × 10^20^ *cm*^−3^) due to field asymmetry and minimization of 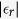. Thus, amplitude modulation with *L*_3*dB*_ = 622 nm can be obtained for Δ*V* = 2.32 V (biasing conditions 1 and 2 on Fig. 4a). The modulator energy-per-bit (*E*), defined by |*E*_*OFF*_ − *E*_*ON*_|, is calculated to be 14.8 fJ. For direct comparison against previously proposed designs, the energy consumed along the extended contact regions is not incorporated into the calculation. Moreover, the experimental power budget will likely be higher, since the exact doping level of as-deposited ITO may be difficult to control and a DC bias will be required to offset the deviation. Nonetheless, the coupled-waveguide architecture, which comprised of two ITO films that each require biasing, shows a 47% reduction in *E* compared against that of a single HPW^11^ (Table I). Note that although complete field symmetry is designed for *V*_*g*_ = 0 V, the waveguide loss decays exponentially as *V*_*g*_ deviates away from 2.32 V in both directions. This is because once 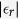 has shifted away from its minimum, the optical power overlapping the ITO accumulation layers becomes minimized and is redistributed into the undoped ITO, *HfO*_2_, and Si layers. Thus, with limited LMI, field symmetry created by manipulating the thickness of other material layers can still be preserved, despite the difference in ITO’s 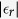 at higher and lower *V*_*g*_. Naturally, propagation loss decreases at a faster rate for lower *V*_*g*_ since free carrier absorption is weaker in the low *n*_*acc*_ regime (see Supplementary Fig. S1).

Typically, the bandwidth (*f*_3*dB*_) of ITO-assisted modulators is dictated by the RC constant of the device instead of the formation time of the accumulation layer inside ITO^11^,^14^,^16^,^17^. Here we assume contact and wiring resistance of 500 Ω^11^ and define *f*_3*dB*_ as 1/RC^11^,^14^. This leads to calculated *f*_3*dB*_ of 363 GHz, comparing favorably against the ~70 GHz reported in previous ITO-modulator that also utilized 5 nm HfO_2_ as gate dielectric^17^. It can be anticipated that the experimental *f*_3*dB*_ and power consumption will also be limited by disorder in the deposited films, additional parasitics from the extended waveguide regions, wiring, and driver electronics. However, utilizing gate dielectric with smaller 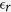 may allow further improvement in our modulator *f*_3*dB*_^11^,^15^. On the other hand, the wavelength dependence of the modulator is displayed in Fig. 4b. In the ON-state, loss can be maintained below 0.05 dB/*μ*m from *λ* = 1300 nm to 1700 nm, further elucidating the robustness and athermal behavior of a coupled-waveguide structure that has engineered field symmetry. On the other hand, in the OFF-state, the waveguide loss is dictated by the ENZ effect and thus is wavelength-sensitive with maximum loss at *λ* = 1535 nm and 3-dB bandwidth of ~270 nm.

Another unique advantage of designing modulators via the two-step methodology is that phase modulation can take place without incurring additional loss. Due to plasma dispersion effect, electrical tuning of carrier concentration in ITO will inevitably leads to change in both material absorption and refractive index. However, in a coupled waveguide structure where long-range propagation is sustained except under the disturbance of the ENZ effect, waveguide loss decays exponentially and nearly symmetrically away from the ENZ point while the change in *n*_*eff*_ is antisymmetric (Fig. 4a). This allows two biasing points on either side of the ENZ peak to have large contrast in *n*_*eff*_ but correspond to identical loss (biasing conditions 3 and 4 on Fig. 4a), which is the necessary condition for phase modulation. Specifically, *δn* of 0.14 and IL = 2 dB/*μ*m can be obtained when the waveguide is biased between *V*_*g*_ = 2 V and 2.75 V. The corresponding interaction length for full-phase modulation is only 5.53 *μm*, with *V*_*π*_*L*_*π*_ of 4.15 V*μ*m, E of 13.8 fJ, and theoretical operating *f*_3*dB*_ of 40.9 GHz.

In addition to amplitude and phase modulation, independent biasing of the ITO layers can reconfigure the waveguide to support complex, coherent modulation schemes. With *V*_*a*_ ≠ *V*_*c*_, it is now possible to determine multiple pairs of *V*_*c*_'s, one for each value of *V*_*a*_, that lead to similar waveguide loss. Concurrently, the difference in *V*_*a*_ will alter the field distribution within the waveguide and therefore the different pairs of gating conditions will be associated with different *n*_*eff*_ values. This offers a path for generating the constellation points required for QAM. For example, four pairs of gating conditions are identified and labeled in Fig. 4c,d, corresponding to identical waveguide loss of ~0.32 dB/*μ*m. At the same time, the associated *n*_*eff*_ values differ significantly and are separated by an uniform Δn of 0.0218. As a result, the four constellation points required for 4-QAM can be generated after light has propagated though a 17.78 *μ*m long waveguide. This is a simple example of how this architecture can extend the amplitude and phase modulation into coherent modulation formats. As shown in Fig. 4e,f, numerous other combinations of gating conditions are available and could also be utilized to compensate for the difference in coupling loss for each constellation point. By utilizing additional permutations of biasing conditions, an array of higher-order modulation formats could be addressed in the same fashion, confirming the degree of versatility of this architecture.

## Discussion

In summary, we reported an architecture for implementing optical modulators that can fully and effectively utilize the tunable properties of ITO. Utilizing an interplay of ENZ effect and field symmetry engineering, hybrid plasmonic waveguide platform with enhanced LMI but minimal loss was realized. This in turn led to integrated modulators with previously unattainable ER-IL ratio, nanoscale footprint, and fJ-level power consumption. Moreover, with a triode-based biasing strategy, it is possible to dynamically induce changes to either the propagation loss or *n*_*eff*_ of a waveguide, thus allowing amplitude, phase, or coherent modulation to be achieved within a single device. As the proof-of-concept, we have only examined how the thickness of the top Si layer and the optical properties of ITO can influence modulation characteristics. These results demonstrated athermal and fabrication tolerant performance. However, further optimization of the properties of other waveguide layers may lead to additional performance enhancement.

It is essential to highlight that field symmetry engineering does not require the two waveguides within the coupled system to be identical. For example, Supplementary Fig. S4 demonstrates that, in a highly asymmetric waveguide structure consisting of Si/ITO/HfO_2_/Al/SiO_2_/Ge stack where only one layer of active material is utilized, IL and modulator FOM can still be minimized and maximized respectively. Thus, high-performance modulators can be designed with great flexibility to suit a wide range of platforms and fabrication processes.

More importantly, the architecture may be utilized for optical modulators that are integrated with other TCOs^10^,^18^, polymers^28^,^29^,^30^, metamaterial^31^, gallium nitride^32^,^33^, or graphene^34^,^35^,^36^,^37^,^38^,^39^,^40^,^41^,^42^,^43^,^44^,^45^,^46^,^47^. These active material platforms are often integrated with plasmonic structure for LMI enhancement and thus could benefit from IL mitigation. In particular, the tunability and ENZ effect within a monolayer graphene, which has thickness comparable to that of the accumulation layer in ITO, has been extensively studied and could utilize our design approach. However, it should be pointed out that the possibility of ENZ effect has recently been a subject of debate due to the anisotropy nature of graphene^48^. If the optical modal field could be engineered to align with the in-plane tunable optical conductivity of graphene, a coupled-plasmonic waveguide modulator may offer ER = 22.5 dB/*μ*m, IL = 0.05 dB/*μ*m, FOM = 450, E = 0.04 fJ, and bandwidth of 11.7 THz (see Supplementary Fig. S5). Overall, our modulator architecture could serve as a platform for implementing thin-film-integrated, travelling-wave modulators, with ER comparable to plasmonic-based designs, IL attainable only in dielectric-based designs, and dynamic reconfigurable modulation formats.

## Methods

### Optical properties of carrier-injected ITO

The permittivity of ITO can be described by the Drude model as^9^:
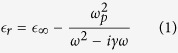
where 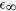 = 3.9 is the high-frequency dielectric constant, *γ* = 1.8 × 10^14^ is the electron scattering rate, *ω*_*p*_ is the plasma frequency, and *ω* is the frequency of light. The plasma frequency term depends on electron concentration (*n*) and is given by:
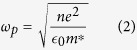
where m^*^ = 0.35 *m*_0_ is the electron effective mass, and *e*, 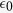, and *m*_0_ are the fundamental charge, the permittivity of free space, and the mass of electron respectively.

### Simulation set-up

The loss and effective index of the optical modes were calculated via two-dimensional finite element method simulations using commercial Lumerical Mode Solutions software. Metallic boundary conditions were utilized to terminate the 2 *μ*m × 2 *μ*m computational domain. Grid size of 0.1 nm, 1 nm, and 2.5 nm were used to mesh the 1 nm accumulation layers, the undoped ITO, HfO_2_, and Al layers, and the rest of the waveguide structure respectively.

The simulations were performed at free space wavelength of *λ* = 1550 nm. The refractive indices of the materials used in mode calculations were as follow: *n*_*Si*_ = 3.47, 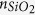 = 1.44, 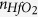 = 1.98, and *n*_*Al*_ = 1.44 + 16i. The optical properties of ITO were evaluated using the Drude formula and plotted in Supplementary Fig. S1.

### Relationship between ITO’s carrier density and gate voltage

The carrier concentration in ITO’s accumulation layer can be described as a function of applied gate voltage (*V*_g_) by the following expression^17^:
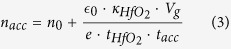
where 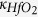 = 25 and 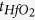 = 5 nm are the DC permittivity and thickness of the *HfO*_2_ gate dielectric, *t*_*acc*_ is the accumulation layer thickness, and *e* and 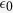 are the fundamental charge and the permittivity of free space respectively. We have assumed *t*_*acc*_ of 1 nm in this report.

## Additional Information

**How to cite this article**: Lin, C. and Helmy, A. S. Dynamically reconfigurable nanoscale modulators utilizing coupled hybrid plasmonics. *Sci. Rep.* **5**, 12313; doi: 10.1038/srep12313 (2015).

## Supplementary Material

Supplementary Information

## Figures and Tables

**Table 1:** Comparison between amplitude modulation performance achieved by recently reported ITO-assisted modulators.

**Device Type**	**t**_**acc**_	**ER**	**IL**	**FOM**	E*
	(nm)	(dB/*μ*m)	(dB/*μ*m)	(ER/IL)	(fJ)
Si nanowire^9^	1	0.11	0.003	37.3	1300
Hybrid plasmonic waveguide^11^	10	1	0.04	25	28
Hybrid plasmonic waveguide^12^	10	6	0.7	8.57	**-
TiN/Cu slot waveguide^17^	3	3.95	0.88	4.5	400
Au slot waveguide^14^	1	2.71	0.45	6.02	***4
**Our design**	**1**	**4.83**	**0.03**	**161**	**14.8**

*Energy-per-bit is calculated using 1/4*CV*^2^.

**The energy-per-bit for Ref. [12] was not reported.

***A low-*κ* gate dielectric was utilized.
