# Supplementary material for: Dynamically reconfigurable nanoscale modulators utilizing coupled hybrid plasmonics
Source: Sci Rep. 2015 Jul 20;5:12313. doi: 10.1038/srep12313 (PMC4507171; doi:10.1038/srep12313)
Supplement: Supplementary Information [file srep12313-s1.pdf]

Supplementary Information:

Dynamically reconfigurable nanoscale modulators utilizing coupled hybrid plasmonics

Charles Lin and Amr S. Helmy

The Edward S. Rogers Department of Electrical and Computer Engineering, University of Toronto, 10 King's College Road, Toronto, Ontario M5S 3G4, Canada

1. **Tunable optical properties of ITO and their effect on the attributes of various plasmonic waveguides.**

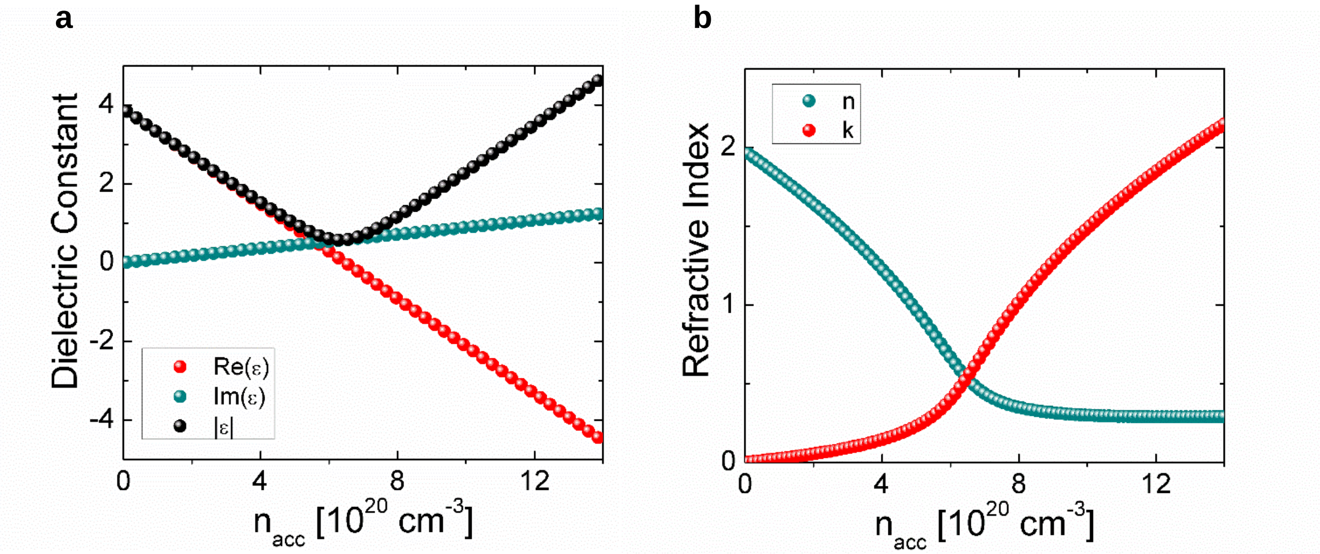

Supplementary Fig. 1 | Optical properties of ITO accumulation layer. a. Dielectric constant (ε_r) and **b.** refractive index of ITO accumulation layer as a function of carrier density (n_{acc}), evaluated using the Drude model [1] at λ = 1550 nm. The effective thickness of the accumulation layer is taken to be 1 nm [2]. Note that ITO's $|\varepsilon_r|$ can be tuned by one order of magnitude, changing from 3.87 at n_{acc} = 1.9x10^{19} cm^{-3} to 0.57 at n_{acc} = 6.6x10^{20} cm^{-3}. Moreover, ITO's accumulation layer transitions from dielectric-like to metal-like state at n_{acc} = 6.47x10^{20} cm^{-3}.

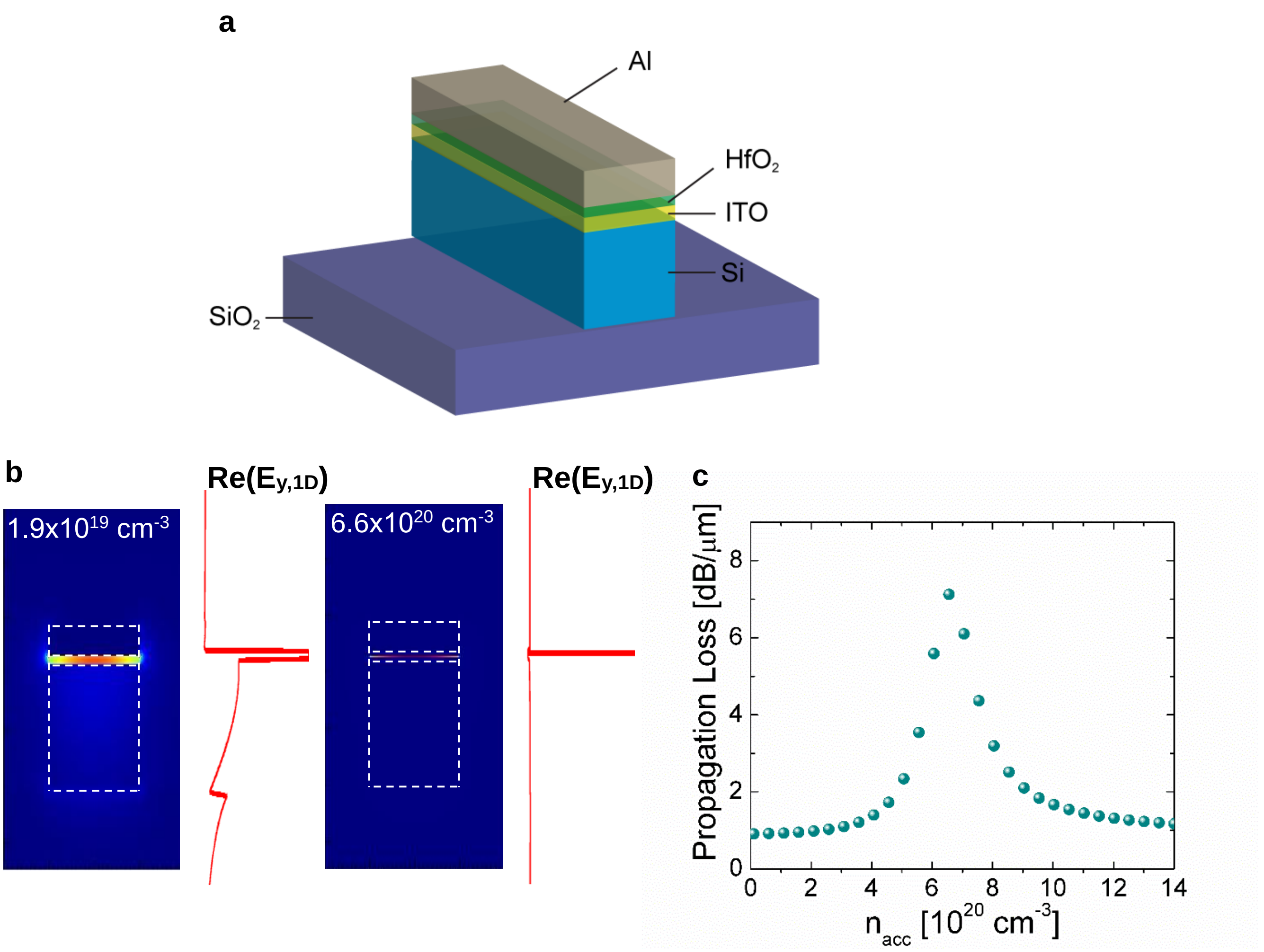

Supplementary Fig. 2 | Characteristics of ITO-assisted hybrid plasmonic waveguide. **a.** Schematic of the waveguide. **b.** Normalized mode profile of the waveguide at ON-state (n_{acc} = 1.9x10^{19} cm^{-3}) and OFF-state (n_{acc} = 6.6x10^{20} cm^{-3}). Note the enhanced light-accumulation layer overlap due to epsilon-near-zero effect in the OFF-state. **c.** Propagation loss of the waveguide as a function of n_{acc} in the accumulation layer. The waveguide loss is 0.91 dB/µm and 7.12 dB/µm at 1.9x10^{19} cm^{-3} and 6.6x10^{20} cm^{-3} respectively. The waveguide width is 200 nm with layers of the following thicknesses: t_{Si} = 220 nm, t_{HfO2} = 5 nm, t_{ITO} = 10 nm, t_{Al} = 50 nm, and $t_{SiO2,substrate}$ = 2 µm.

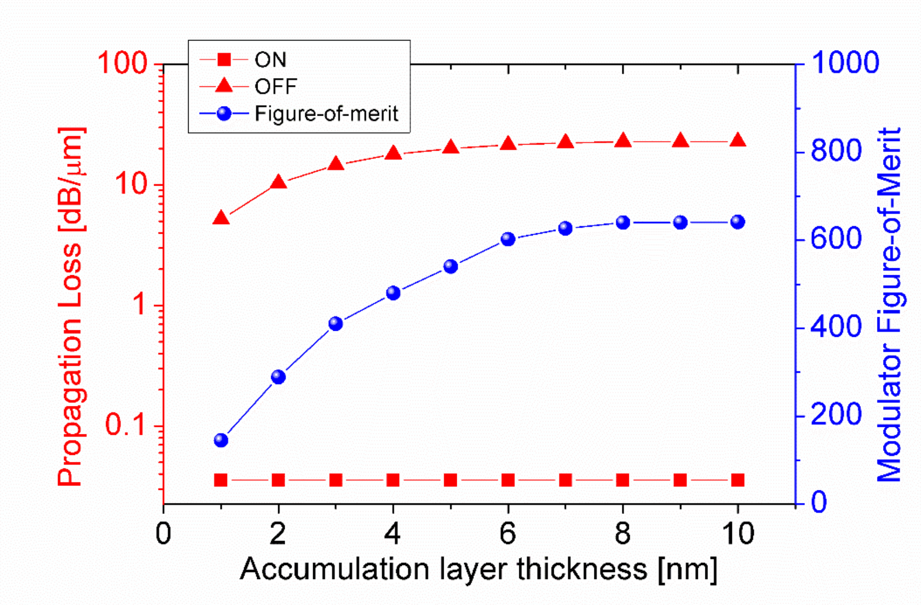

Supplementary Fig. 3 | Effect of accumulation layer thickness on modulation performance. The ON-state (n_{acc} = $1x10^{19}$ cm^{-3}) and OFF-state (n_{acc} = $6.6x10^{20}$ cm^{-3}) propagation loss of symmetric supermode of the ITO-assisted coupled-hybrid plasmonic waveguide are displayed as a function of the thickness of the ITO accumulation layer. The corresponding figure-of-merit (extinction ratio/ insertion loss) is also shown. The ON-state loss is almost independent of accumulation layer thickness. On the other hand, by increasing the accumulation layer thickness, the overlap between the optical mode and ITO is enhanced in the OFF-state, leading to higher figure-of-merit. The waveguide width is 200 nm with layers of the following thicknesses: $t_{Si,bottom}$ = 220 nm, t_{ITO} = 10 nm, t_{HfO2} = 5 nm, $t_{Si,top}$ = 240 nm, t_{Al} = 10 nm, and $t_{SiO2,substrate}$ = 2 μm.

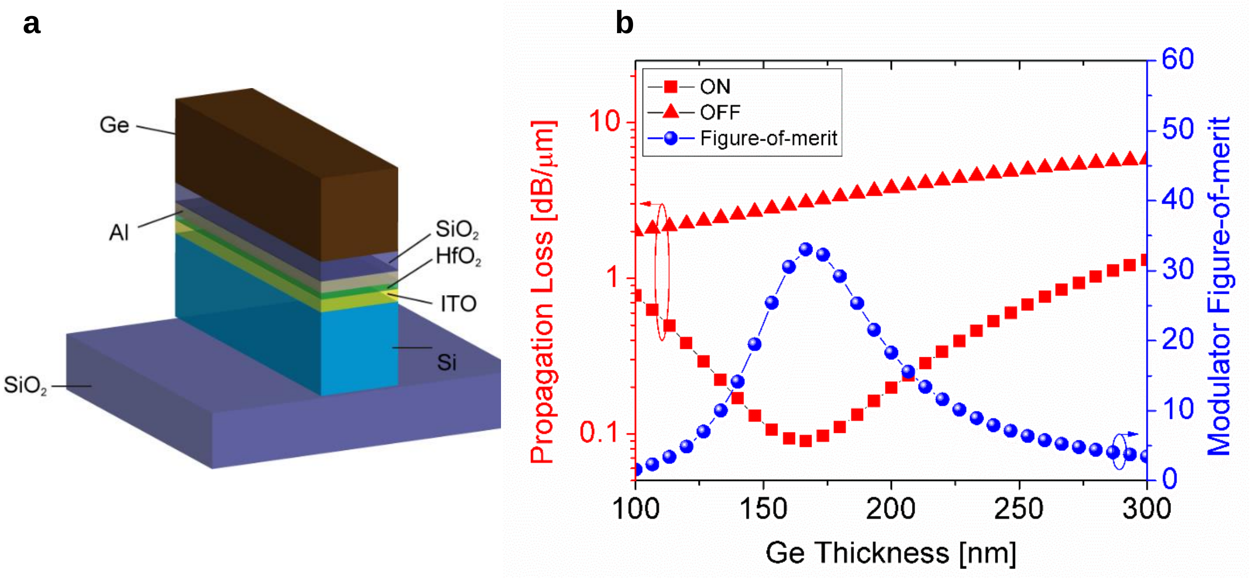

Supplementary Fig. 4 | Asymmetric, ITO-assisted, coupled-hybrid plasmonic waveguide modulator. a. Schematic of the waveguide. **b.** Loss of the symmetric supermode of the asymmetric structure as a function of the thickness of the top Ge layer. Although the waveguide consists of two non-identical hybrid plasmonic waveguides and comprised of only a single active ITO layer, loss minimization in the ON-state can still be achieved by manipulating structural parameters to engieer a symmetric field distribution across the metal. The waveguide width is 200 nm with layers of the following thicknesses: t_{Si} = 220 nm, $t_{SiO2,spacer}$ = 15 nm, t_{HfO2} = 5 nm, t_{ITO} = 10 nm, and t_{Al} = 10 nm. The refractive index of Ge at 1550 nm is taken to be 4.275+0.00567i.

2. Graphene-assisted coupled-hybrid plasmonic waveguide modulator

The graphene-assisted couple-hybrid plasmonic waveguide configuration is illustrated in Supplementary Fig. S5a. The tunable optical properties of the two monolayer graphene sheets, each embedded within a hexagonal boron nitride (hBN) spacer, can be described by Kubo's formula as a function of chemical potential (μ) (Supplementary Fig. S5b) [3]. Graphene's substrate-dependence is not considered in this work since the in the near-infrared range, the optical properties of a free-standing monolayer graphene is retained when placed onto a hBN substrate [4,5]. Moreover, the substrate-induced shifts in optical conductivity of graphene could be compensated through additional DC bias. Thus, substrate-induced changes will not affect the validity of utilizing coupled-waveguide structure to achieve improved modulator performance. Similar to the case of ITO-assisted design, by electrically inducing graphene to be in the ENZ state, amplitude, phase, and coherent amplitude modulation could be obtained (Supplementary Fig. S5d - S5g).

The fabrication of this modulator configuration requires multiple electron-beam-lithography patterning, film deposition/transfer, and plasma etching steps. To reduce transfer-related defects, graphene and hBN films can first be grown either via mechanical exfoliation or chemical vapor deposition, assembled into hBN/graphene/hBN heterostructures via the van der Waals assembly technique, and subsequently transferred onto silicon-on-insulator substrate to be patterned [6-8]. Hydrogenated amorphous polysilicon can be deposited via low temperature-PECVD and serves as the top high-index dielectric layer [9]. To minimize the disturbance to the optical mode, contacts will be fabricated on narrow strips of Al and graphene films that have extended several hundred nanometers away from the waveguide. The edges of encapsulated graphene will be exposed through plasma etching, followed by deposition of Cr/Pd/Au layers to make edge contacts [6,7,10].

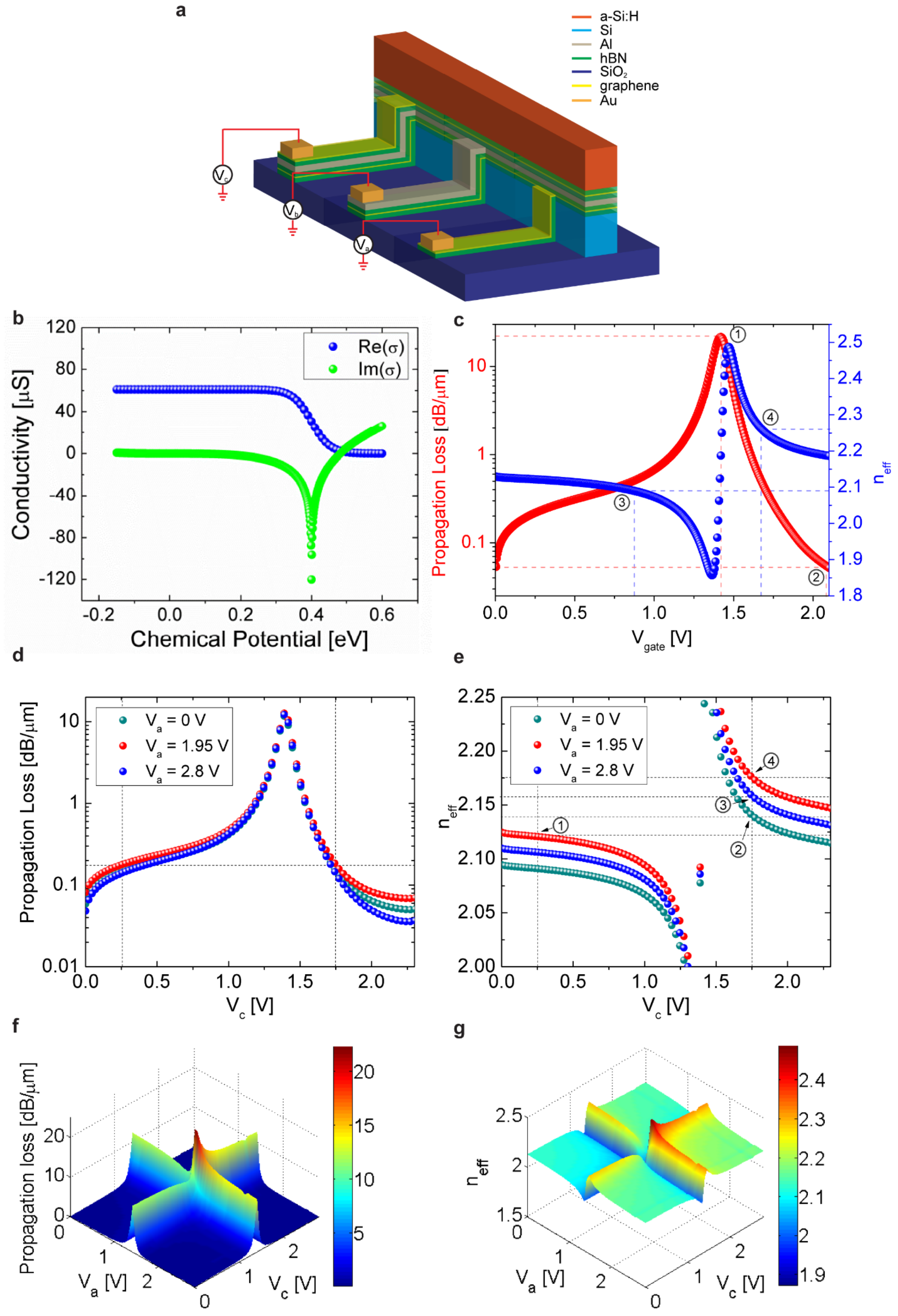
a
a-Si:H
Si
Al
hBN
SiO2
graphene
Au
Vc
Vb
Va
b
Re(σ)
Im(σ)
Conductivity [μS]
Chemical Potential [eV]
c
Propagation Loss [dB/μm]
neff
Vgate [V]
d
Va = 0 V
Va = 1.95 V
Va = 2.8 V
Propagation Loss [dB/μm]
Vc [V]
e
Va = 0 V
Va = 1.95 V
Va = 2.8 V
neff
Vc [V]
f
Propagation loss [dB/μm]
Va [V]
Vc [V]
g
neff
Va [V]
Vc [V]

Supplementary Fig. 5 | Modulation performance of graphene-assisted coupled-HPW modulator at λ = 1550 nm. a. Schematic of the waveguide. **b.** The dielectric constant (ε) of monolayer graphene as a function of chemical potential (μ) at λ = 1550 nm, calculated from the Kubo formula [3] with effective thickness of 0.7 nm [11]. |ε| can vary rapidly from a maximum of 17.4 at μ = 0.4 eV to a minimum of 0.126 at μ = 0.513 eV. **c.** Propagation loss and n_{eff} of the waveguide as a function of V_{gate} (V_a=V_c=V_{gate}). The relation between μ and V_{gate} can be calculated using a parralel plate capacitor model [11]. Amplitude modulation can be achieved using biasing conditions 1 (V_{gate} = 1.42 V) and 2 1 (V_{gate} = 2.1 V), which provides ER = 22.5 dB/μm, IL = 0.05 dB/μm, FOM = 450, and E = 0.04 fJ. We assume sheet resistivity of graphene and total edge contact resistance to be 40 Ω/□ and 400 Ω respectively [6], both of which are experimentally measured and thus account for the influence of the hBN substrate on graphene's charge carrier mobility. This leads to a theoretical operating bandwidth of 11.7 THz for 3-dB amplitude modulation within the waveguide section. The experimental modulator bandwidth will be a fraction of the theoretical value due to crystalline disorder of graphene and the excess parasitics from contact region and driver electronics. Phase modulation can be obtained using biasing conditions 3 (V_{gate} = 0.86 V) and 4 (V_{gate} = 1.67 V), corresponding to Δn_{eff} of 0.17, IL = 0.5 dB/μm, and full-phase modulation length of 4.55 μm. **d.** Propagation loss and **e.** n_{eff} of the waveguide as a function of V_c for different V_a's. For V_c = 0.25 V or 1.75 V, waveguide losses are nearly identical but the corresponding n_{eff} vlaues differ significantly and are separated by an uniform Δn of 0.0154. As a result, the four constellation points required for 4-QAM (using biasing conditions 1 to 4) can be generated within a single waveguide with length of 22.4 μm. **f.** Propagation loss and **g.** n_{eff} of the waveguide as a function of both V_a and V_c. V_b is grounded in all cases.

References

[1] Vasudev, A. P., Kang, J., Park, J., Liu, X., & Brongersma, M. L. Electro-optical modulation of a silicon waveguide with an "epsilon-near-zero" material. *Opt. Express* **21,** 26387-26397 (2013).

[2] Krasavin, A. V. & Zayats, A. V. Photonic signal processing on electronic scales: electro-optical field-effect nanoplasmonic modulator. *Phys. Rev. Lett.* **109,** 053901 (2012).

[3] Stauber, T., Peres, N. M. R., & Geim, A. K. Optical conductivity of graphene in the visible region of the spectrum, *Phys. Rev. B* **78,** 085432 (2008).

[4] Principi, A. *et al.* Plasmon losses due to electron-phonon scattering: The case of graphene encapsulated in hexagonal boron nitride. *Phys. Rev. B* **90,** 165408 (2014).

[5] Lin, X. *et al.* Ab initio optical study of graphene on hexagonal boron nitride and fluorographene substrates. *J. Mater. Chem. C* **1,** 1618{1627 (2013).

[6] Wang, L. *et al.* One-Dimensional Electrical Contact to a Two-Dimensional Material. *Science* **342,** 614--617 (2013).

[7] Gao, Y. *et al.* High-Speed Electro-Optic Modulator Integrated with Graphene-Boron Nitride Heterostructure and Photonic Crystal Nanocavity. *Nano Lett.* **15,** 2001--2005 (2015).

[8] Kim, K., Choi, J., Kim, T., Cho, S., & Chung, H. A role for graphene in silicon-based semiconductor devices. *Nature* **479,** 338344 (2011).

[9] Harke, A., Krause, M., & Mueller, J. Low-loss singlemode amorphous silicon waveguides. *Electron. Lett.* **41,** 1377--1379 (2005).

[10] Smith, J. T., Franklin, A. D., Farmer, D. B., & Dimitrakopoulos, C. D. Reducing Contact Resistance in Graphene Devices through Contact area Patterning. *ACS Nano* **7,** 3661--3667 (2013).

[11] Liu, M. et al. A graphene-based broadband optical modulator. *Nature* 474, 64-67 (2011).
